# Transcription factor EB (TFEB) improves ventricular remodeling after myocardial infarction by inhibiting Wnt/*β*-catenin signaling pathway

**DOI:** 10.7717/peerj.15841

**Published:** 2023-08-18

**Authors:** Cong Liu, Dawang Zhou, Qiang Zhang, Hongyan Wei, Yuanzheng Lu, Bo Li, Haohong Zhan, Jingge Cheng, Chuyue Wang, Yilin Yang, Shuhao Li, Chunlin Hu, Xiaoxing Liao

**Affiliations:** 1 Department of Emergency Medicine, The Seventh Affiliated Hospital, Sun Yat-sen University, Shenzhen, China; 2 Department of Emergency Medicine, The First Affiliated Hospital, Sun Yat-sen University, Guangzhou, China

**Keywords:** Myocardial infarction, Transcription factor EB, Ventricular remodeling

## Abstract

**Background:**

Adverse left ventricular remodeling after myocardial infarction (MI) compromises cardiac function and increases heart failure risk. Until now, comprehension of the role transcription factor EB (TFEB) plays after MI is limited.

**Objectives:**

The purpose of this study was to describe the effects of TFEB on fibroblasts differentiation and extracellular matrix expression after MI.

**Methods:**

AAV9 (adeno-associated virus) mediated up- and down-regulated TFEB expressions were generated in C57BL/6 mice two weeks before the MI modeling. Echocardiography, Masson, Sirius red staining immunofluorescence, and wheat germ agglutinin staining were performed at 3 days, and 1, 2, and 4 weeks after MI modeling. Fibroblasts collected from SD neonatal rats were transfected by adenovirus and siRNA, and cell counting kit-8 (CCK8), immunofluorescence, wound healing and Transwell assay were conducted. Myocardial fibrosis-related proteins were identified by Western blot. PNU-74654 (100 ng/mL) was used for 12 hours to inhibit *β*-catenin-TCF/LEF1 complex.

**Results:**

The up-regulation of TFEB resulted in reduced fibroblasts proliferation and its differentiation into myofibroblasts in vitro studies. A significant up-regulation of EF and down-regulation of myocyte area was shown in the AAV9-TFEB group. Meanwhile, decreased protein level of *α*-SMA and collagen I were observed in vitro study. TFEB didn’t affect the concentration of *β*-catenin. Inhibition of TFEB, which promoted cell migration, proliferation and collagen I expression, was counteracted by PNU-74654.

**Conclusions:**

TFEB demonstrated potential in restraining fibrosis after MI by inhibiting the Wnt/*β*-catenin signaling pathway.

## Introduction

Acute myocardial infarction (AMI) is caused by hypoxia and ischemia, with high morbidity and mortality (Bai et al., 2021). AMI often leads to heart failure (HF), which is the major risk patients have to face (Bahit, Kochar & Granger, 2018; Velagaleti et al., 2008). Therefore, effective treatment is needed to reduce the size of myocardial infarction (MI), preserve left ventricular (LV) function, and prevent HF in patients with AMI. After AMI, extensive myocardial injury, impaired myocardial contractility, continuous activation of the neuroendocrine system, and remodeling of extracellular matrix (ECM) occur in the heart, which results in left ventricular remodeling (LVR). Adverse left ventricular remodeling affects cardiac function and increases the risk of HF (Azevedo et al., 2016). Nowadays, myocardial remodeling is recognized as a complex process in response to cardiac overload and loss of functional myocardium, resulting in structural and functional disorders of the myocardium (Van der Bijl et al., 2020).

The post-AMI cardiac response can be divided into three phases: (1) a proinflammatory phase (days 1–3 after MI) aimed at removing cellular debris from the ischemic infarct zone. In the ischemic environment, myocardial cells undergo anaerobic metabolism, cell membrane is unstable, and cells undergo apoptosis, autophagy and necrosis (Fu et al., 2020; Heusch & Gersh, 2017). Neutrophils and macrophages lead to destruction of the extracellular collagen matrix (ECM) and enlargement of the infarct area, thereby changing the shape of the ventricle, and the infarct myocardium becomes thinner and dilated. (2) During the repair period (4–7 days after MI), acute inflammatory mechanisms are down-regulated and myocardial injury is alleviated, while wound healing and scar formation are performed to prevent cardiac rupture. After the inflammatory response, fibroblasts are directed to the infarct area, where they produce new collagen matrix and form scar tissue (Bhatt, Ambrosy & Velazquez, 2017; Gabriel-Costa, 2018; Sutton & Sharpe, 2000; Xie, Burchfield & Hill, 2013). (3) At the mature stage (7 days after MI), the non-infarcted cardiomyocytes became hypertrophic and the ECM changed. The cardiac ECM is a highly organized structural and functional protein network that surrounds cardiomyocytes and generates a cellular scaffold that maintains LV shape (Iyer et al., 2014). If inflammation and fibrosis-related signals are continuously activated, it may lead to adverse left ventricular remodeling (Ong et al., 2018; Ruparelia et al., 2017; Westman et al., 2016).

After MI, fibroblasts will proliferate and differentiate into myofibroblasts. Myofibroblasts express the contractile proteins *α*-smooth muscle actin (*α*-SMA) and embryonic smooth muscle myosin, exhibit an extended endoplasmic reticulum, and secrete abundant matrix proteins to generate collagen scars (Frangogiannis, Michael & Entman, 2000). Fibroblasts early activation and late remodeling is important for cardiac function (Van Nieuwenhoven & Turner, 2013). Adverse fibrosis will lead to myocardial stiffness, diastolic and systolic dysfunction, and eventually the development of HF (Prabhu & Frangogiannis, 2016).

TFEB is one member of the microphthalmia-associated transcription factor E family and is actively involved in many cellular biological and pathological processes (Rehli et al., 1999). TFEB studies showed that under starvation or stress conditions, TFEB dephosphorylated and accumulated in the nucleus, recognized and bound to genes sequence, upregulated genes expression at the transcriptional level involved in lysosome and autophagosome biogenesis, as well as phagocytosis and various immune responses (Godar et al., 2015; Ma et al., 2015; Medina et al., 2011; Palmieri et al., 2011; Settembre et al., 2011). Until now, whether TFEB was involved in the fibrosis process after MI remains unclear. Thus, we designed this study to systematically evaluate the impacts of TFEB on the pathology processes of autophagy, ventricular remodeling, and fibrosis after MI.

The Wnt signaling is involved in cell proliferation and differentiation progress, and is necessary for cardiac myocyte formation (Chen et al., 2001; Moon et al., 2002). Extracellular Wnts bind to transmembrane receptor complexes. The destruction complex consisting of *β*-catenin, Axin, APC (adenomatous polyposis coil), CK1 (casein kinase1) and GSK3 (glucogen synthase kinase 3 beta), has been relocated to the plasma membrane. The *β*-catenin is free from the destruction complex and accumulates within the cytosol. And this leads to stabilisation and translocation of *β*-catenin into the nucleus, where it binds to T-cell factor (TCF) and the lymphoid enhancer factor (LEF), and activates various target genes (Clevers, 2006; Moon et al., 2004). Studies show that the Wnt signaling pathway is involved in the fibroblast activation and proliferation during cardiac fibrosis (Duan et al., 2012; Honda et al., 2013; Sullivan & Black, 2013).

Here, we showed that TFEB, a transcription factor related to autophagy and lysosomal biogenesis, is involved in the progression of fibrosis. We also found that the inhibition effect of TFEB in fibrosis was mediated by the formation TFEB- *β*-catenin-TCF/LEF1 complex, which changed the gene expression profile of *β*-catenin.

## Materials and Methods

### Reagents

Portions of this text were previously published as part of a preprint (Liu et al., 2022). TGF-ß1 was purchased from Sino Biological (Beijing, China). The adeno-associated virus 9 (AAV9) was purchased from Weizhen Technology Co., LTD. (Shandong, China). In the TFEB-overexpression mice built by adeno-associated virus, gene ID: NM_011549, vector: PAV-CMV-P2A-GFP (CMV promoter), virus titer: 3.84 × 10^13^ µg/ml; AAV9-NC was GFP (CMV promoter) control adeno-associated virus with a titer of 8.02 × 10^13^ µg/mL. In the AAV9-shTFEB mice with TFEB expression down-regulated by AAV9 4in1shRNA, gene ID: NM_011549, vector: PAV-4IN1shrNA-GFP; primer design: positive CGGCAGTACTATGACTATGA, reverse: GCCGTCATGATACTGATACTA; Virus titer: 2.22 × 10^13^ µg/mL; AAV9-sh-TFEB virus was AAV9-U6-GFP control adeno-associated virus inserted with nonsense sequence, and the virus titer was 4.74 × 10^13^ µg/mL. All the dilution virus titer was 1 × 10^13^ ug/ml, and the dose was 10 µl for each mouse. The adenovirus used in the *in vitro* study was purchased from Hanheng Biology (Shanghai, China). Ad-TFEB was HBAD-TFEB-EGFP overexpressed virus, gene sequence number: M_001025707.  Ad-GFP was used as a control virus with HBAD-EGFP overexpression. The multiplicity of cellular infection (MOI) of the virus infection complex in the 6-well plate was 30, with an adenovirus volume of 10 µL. The MOI of the virus infection complex in the 24-well plate was 30, with an adenovirus volume of 3 µL. The siRNAs were used to reduce TFEB expression, with siNC serving as control. The target sequence of siRNAs: GCAGTCTCAGCATCAGAAA.

### Ethical approval

In this study, all animal experiments were carried out in accordance with the ARRIVE guidelines and were approved by the Institutional Animal Care and Use Committee of the Sun Yat-Sen University(SYSU-IACUC-2021-000659).

### Animals and MI modeling

**Figure 1:**
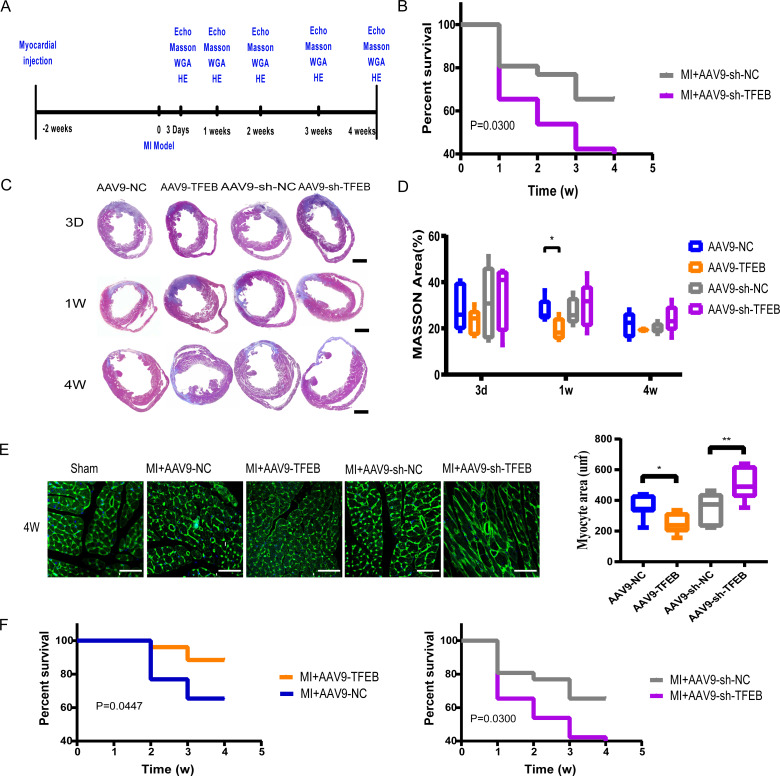
Extent of MI, myocardial hypertrophy and systolic function of heart. (A) Procedure of mice MI modeling after injecting with AAV9. (B) Echocardiography of the mice and the qualified EF ratios in each group. (C) MI areas display in the Masson-stained mouse hearts. Scale bars represent one mm. (D) Ratios of MI areas to the ventricular cavity. (E) Myocyte areas four weeks after MI modeling in different groups (qualified from the WGAs staining). Scale bars represent 50 µm. (F) Survival curves of mice in each group (*: *p* < 0.05, **: *p* < 0.01).

We used male mice to establish a stable MI model considering that estrogen has shown a protective effect against pathological hypertrophic remodeling in pressure-overload (Cao et al., 2011; Patten et al., 2004). Two-month-old wild-type male C57BL/6 mice (20-30g) were purchased from the Animal Experimental Centre of Jicui Yaokang Biotechnology Co. LTD. (Jiangsu, China). All mice were fed in the SPF animal laboratory at the Animal Center of Sun Yat-Sen University, Guangzhou, China, with free access to standard laboratory food and water. 142 mice were used to construct MI models and 130 mice were included. 12 mice were excluded because of technical failure. 130 mice were randomized into five groups: the sham group, AAV9-TFEB group, AAV9-NC group, AAV9-shTFEB group, and the AAV9-shNC group, with random allocation software by Dr. Cong Liu. One, three, five, nine and six mice of each group died after MI. The AAV9-NC and AAV9-sh-NC group served as controls of the AAV9-TFEB and AAV9-sh-TFEB groups, respectively. Every mouse was pretreated with myocardial multipoint injection of Adeno-associated virus 9 (AAV9) or normal saline for two weeks before the MI modeling. After 14 days of feeding, the anterior descending branch of the mice’s left coronary artery (LAD) was permanently ligated to set up the MI model. The surgery was conducted under the anesthetization of 50 mg/kg intraperitoneally injected pentobarbital sodium (Sigma). For mice in the sham group, the chest was surgically opened without LAD ligation. Echocardiography was performed every time point. The mice were anesthetized and sacrificed by cervical dislocation at different time points after MI or sham operation. Hearts were collected and stored at −80 °C for the next step measurements. The flowchart of this experiment is illustrated in Fig. 1A. Each test was repeated with at least three mice in each group. Each test was performed by the same person.

### Echocardiography

Echocardiography was performed on the VisualSonics machine Vevo 3100. Mice were anesthetized by Isoflurane (Rayward Life Technology Co., LTD., Shenzhen, China). Mouse chests were hair-shaved, and the animals were positioned on a warm cushion. Left ventricular ejection fraction (LVEF) was measured in M-mode short-axis at the level of papillary muscles.

### Masson staining, Sirius red staining and Wheat germ agglutinin staining

The infarction regions of left ventricular tissues were fixed in 4% paraformaldehyde (Servicebio, Wuhan, China) for at least 24 h, then embedded in paraffin. Sections of 3–6 µm thickness were stained following the standard protocol of Masson trichrome (BP028; Biossci, Beijing, China), Picro Sirius Red Stain Kit (Phygene, Fuzhou, China) and fluorescein isothiocyanate-conjugated wheat germ agglutinin (WGA-FITC, MP6325; MKbio, Gyeonggi-do, China) respectively. In Masson-stained sections, myocardial cells appear red, while collagen appears blue. After sirius red staining, collagen I appears orange, and Collagen III appears green. The results of WGA (Wheat germ agglutinin (WGA) staining) staining were observed under a confocal microscope (LSM 880; Zeiss, Oberkochen, Germany).

### Immunofluorescence analysis

In the immunofluorescence staining, the *β*-catenin was tagged by *β*-catenin antibody (1:200; Affinity), and TFEB protein was tagged by TFEB (1:200; Absin) antibody. and the nuclei were counterstained with 0.5 µg/mL 4′,6-diamidino-2-phenylindole (DAPI; 1:500, Solarbio). The result of staining was imaged using an immunofluorescence microscope (BX53; Olympus).

### Protein extraction and Western blotting

Proteins in Cells or mice organs were extracted following standard procedures using the protein extraction reagents of Ripa (Millipore, Burlington, MA, USA), PMSF (CST), a protease inhibitor (Roche), and phosphatase inhibitor (Roche, Basel, Switzerland). The protein concentration was tested by the BCA Quantitative Kit (Thermo Fisher Scientific, Waltham, MA, USA). Equal amounts of total protein (30 µg) were separated by 8% SDS-PAGE gels and transferred to PVDF membranes. After being blocked in 5% skim milk for one hour, the membranes were subsequently incubated with primary antibodies at 4 °C overnight and then the secondary antibodies at room temperature for one hour. The membranes were then exposed to chemiluminescence developing agents. The antibodies used in this process were as followed: mouse anti-Collagen III (NBP1-05119, Novus), rabbit anti-Collagen I(NB600-408; Novus, Zhejiang, China), rabbit anti- *β*-catenin (AF6266; Affinity), rabbit anti-TFEB (abs131998; Absin, Shanghai, China), and rabbit anti-GAPDH (sc-166545; Santa Cruz Biotechnology, Dallas, TX, USA). GAPDH was used as an internal control.

### Myocardial fibroblasts isolation and culture

Primary neonatal rat myocytes were isolated from the heart of 1- to 3-day-old SD rats, digested with 0.05% collagenase type II and trypsin, and dispersed *via* gentle mechanical attrition. After centrifugation, cells were cultured in DMEM/F-12 (Gibco), supplemented with 10% fetal bovine serum (Gibco, Billings, MT, USA), 50 U/mL penicillin, 50 µg/mL streptomycin in a 37 °C, 5% CO2 incubator in NHC key Laboratory of Assisted Circulation. The second generation of the CFs was used for the experiments. Cells were treated with virus or siRNA and were cultured in a serum-free medium at least 24 h before being treated with 5 ng/ml TGF-ß1(Sino Biological, Beijing, China) or in combination with 100 µM PNU-74654 (HY-101130, MCE) for 12 h.

### Cell counting kit-8 (CCK-8) assay and EDU assay

CFs were transferred into 24-well plates. Cells were treated with Virus or siRNAs and were cultured for at least 24 h in a serum-free medium before being treated with 5ng/ml TGF-ß1 for 12 h. The proliferation of cells was determined by a CCK-8 kit (MCE) and an EDU kit (KTA2030; Abbkine). The optical density (OD) of each well was examined at 450 nm using a microplate reader (Thermo Fisher Scientific). The proliferation rate was calculated by the results of OD and fluorescence.

### Transwell assay and wound healing

Transwell assay and wound healing was performed on the second generation of the CFs respectively. Cells were treated with Virus or siRNAs and were cultured for at least 24 h in a serum-free medium before being co-incubated with 5ng/ml TGF-ß1and the Transwell inserts for 12 h. CF cells were plated on the upper side of the chambers in Transwell assay. After 12 hour’s incubation, the cells that migrated to the lower side of the chambers were counted through DAPI staining. Draw a straight line with a 200ul tip in wound assay. Photographs were taken continuously for 24 h by Biotek Lionheart F.

### Statistical analysis

All data were presented as the means ± standard deviation (SD). The results were analyzed by one-way analysis of variance (ANOVA) or the Student *t*-test, and a *p* < 0.05 was considered to be statistically significant.

## Results

### Extent of MI, myocardial hypertrophy and systolic function of heart

To evaluate the effect of TFEB on cardiac function after MI, a MI model was made in mice. Four weeks after MI, left ventricular hypertrophy (Fig. S1). AAV9 was used to up-regulate or down-regulate the expression of TFEB (Fig. S2). The echocardiography after three and four weeks of MI modeling confirmed a higher EF value in the AAV9-TFEB group (Fig. 1B and Fig. S3). In the AAV9-TFEB group, the ratio of infarcted area to left ventricle length was lower than the AAV9-NC group four weeks after modeling (Figs. 1C–1D). The surviving cardiomyocytes become hypertrophy as time prolongs (Figs. S4A–S4C). AAV9-shTFEB group showed a larger myocyte area than the AAV9-shNC group. WGA staining showed that TFEB down-regulation was associated with more severe cardiac hypertrophy (Fig. 1E). Survival curves were significantly better among the AAV9-TFEB group than the AAV9-NC group, and worse among the AAV9-shTFEB group (Fig. 1F) (log-rank *P* < 0.05).

### TFEB affected the differentiation of fibroblasts and extracellular matrix synthesis and transformation *in vivo*

**Figure 2:**
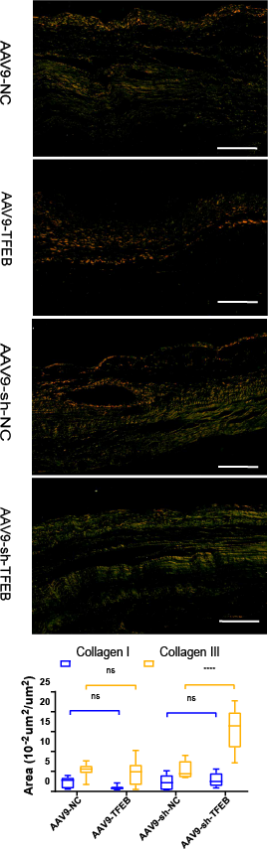
TFEB affected the differentiation of fibroblasts and extracellular matrix synthesis and transformation *in vivo*. MI areas stained by Sirius red four weeks after MI. Collagen I was stained into orange and Collagen III was stained into green. Scale bars represent 10 µm. The expression levels of Collagen I and Collagen III were quantified and presented as mean ± standard deviation. (*: *p* < 0.05, **: *p* < 0.01, ****: *p* < 0.0001).

Two to four days after modeling, fibroblasts activated by the stimulation of inflammatory cytokines began to proliferate and produce ECM (Kurose, 2021). As revealed by the Sirius red staining, AAV9-shTFEB group had a higher concentration of collagen I and III than the AAV9-shNC group (Fig. 2A).

### The effects of TFEB on cardiac fibroblasts during fibrosis model in vitro

**Figure 3:**
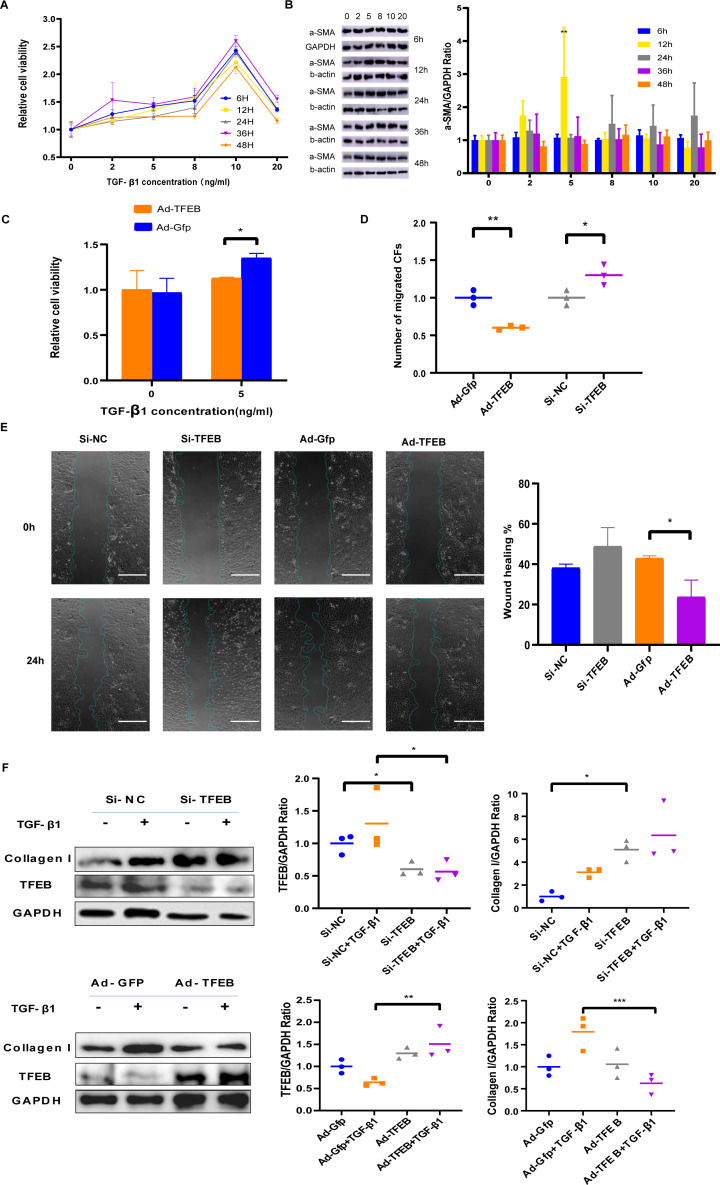
TFEB Affected CFs in Fibrosis model *in vitro*. (A) Effects of TGF- *β*1 of different concentrations and incubation time on proliferation of CFs of rats were measured by CCK-8 assay. (B) The expression of *α*-SMA was detected using western blotting. (C) Cell viability assessed by CCK-8 assay after rat CFs were treated with increasing concentration of TGF- *β*1 for 12 h. (D) Cell migration was evaluated by the Transwell assay for the unstimulated CFs (control) and the CFs co-incubated with TGF- *β*1 (5 ng/mL) for 12 h. (E) Cell migration was evaluated by wound healing assay for the unstimulated CFs (control) and the CFs co-incubated with TGF- *β*1 (5 ng/mL) for 24 h. Scale bars represent 500 µm. (F) The expression difference of Collagen I in CFs was detected using western blot (*: *p* < .05, **: *p* < 0.01, ***: *p* < 0.001, ****: *p* < 0.0001, ##: *p* < 0.01).

TGF-ß1 was used to simulate fibroblast proliferation and its differentiation into myofibroblasts. CFs cells were co-incubated with TGF- *β*1 of different concentrations including 2 ng/mL, 5 ng/mL, eight ng/mL, 10 ng/mL, and 20 ng/mL for different duration including 6, 12, 24, 36, and 48 h. CCK-8 results indicated that low concentration TGF- *β*1 promoted CFs proliferation, while high concentrations weakened the promotion effect (Fig. 3A). Western blot showed that *α*-SMA elevated obviously with 5ng/ml TGF- *β*1 (Fig. 3B). Ad-TFEB group showed lower proliferation rate than the Ad-Gfp group in the CCK-8 test after 12 h of co-incubation with 5ng/ml TGF- *β*1 (Fig. 3C). Transwell and Wound healing results showed the Ad-TFEB group had a lower cell migration than the Ad-Gfp group (Figs. 3D–3E and Fig. S5). Western blot results indicated that 12-hour TGF- *β*1 stimulation increased collagen I expression. The Si-TFEB group had a higher collagen I expression than the Si-NC group (Fig. 3F).

### TFEB relocated to nucleus and made connection with Wnt pathway by *β*-catenin-TCF/LEF1 complex

**Figure 4:**
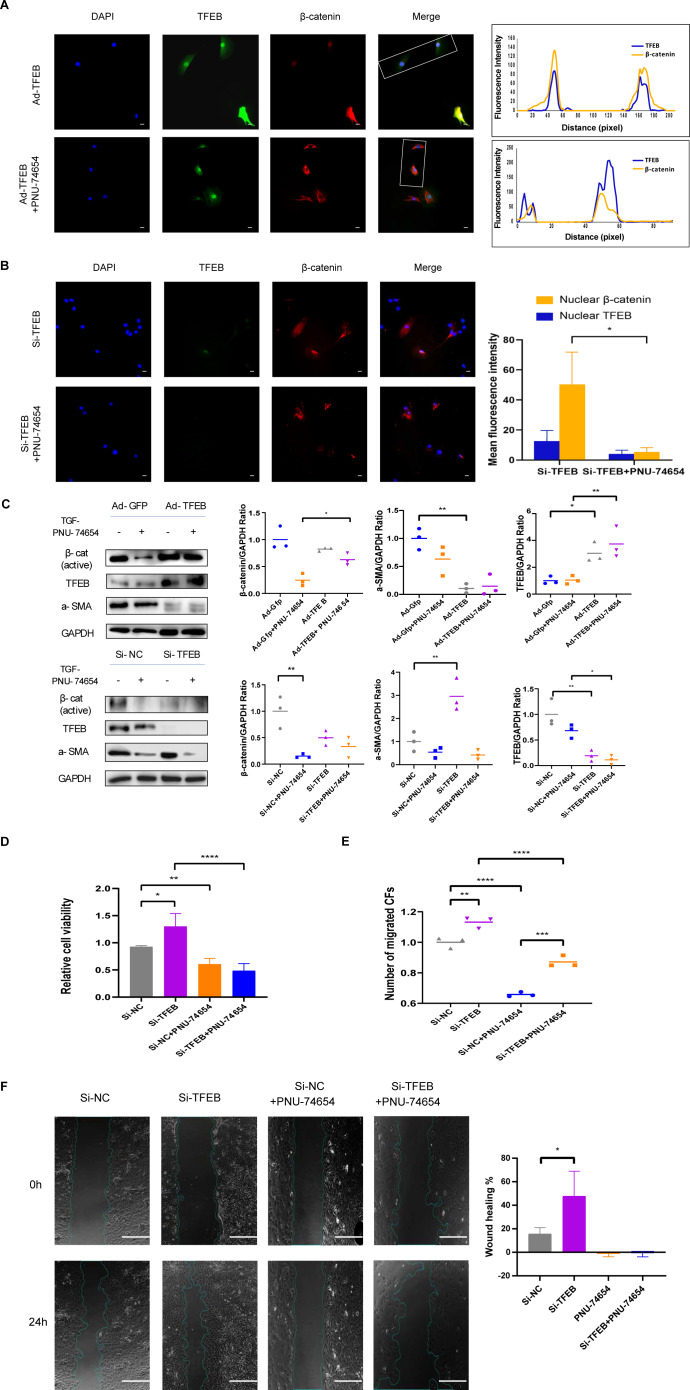
TFEB relocated to nuclear and made connection with Wnt pathway by *β*-catenin-TCF/LEF1 complex. (A) Immunofluorescence staining of TFEB (GFP) and *β*-catenin (red) of the CFs co-incubated with TGF- *β*1 (5 ng/mL) for 12 h. The interaction between TFEB and *β*-catenin was showed using high resolution confocal microscopy analysis s. PNU-74654 (100 ng/mL) was used to for 12 h to inhibit *β*-catenin-TCF/LEF1 complex (40X). Scale bars represent 100 µm. (B) Immunofluorescence staining of TFEB (GFP) and *β*-catenin (red) of the CFs co-incubated with TGF- *β*1 (5 ng/mL) for 12 h. Si-TFEB was used to inhibit TFEB (40X). Scale bars represent 100 µm. (C) The expression of *α*-SMA was detected using western blotting. PNU-74654 (100 ng/mL) was used to for 12 h to inhibit *β*-catenin-TCF/LEF1 complex. (D) Cell viability assessed by CCK-8 assay after rat CFs were treated with TGF- *β*1 (5 ng/mL) for 12 h. PNU-74654 (100 ng/mL) was used to for 12 h to inhibit *β*-catenin-TCF/LEF1 complex. (E) Cell migration was evaluated by the Transwell assay for the CFs co-incubated with TGF- *β*1 (5 ng/mL) for 12 h. PNU-74654 (100 ng/mL) was used to for 12 h to inhibit *β*-catenin-TCF/LEF1 complex. (F) Cell migration was evaluated by wound healing assay for the CFs co-incubated with TGF- *β*1 (5 ng/mL) for 24 h. PNU-74654 (100 ng/mL) was used to for 12 h to inhibit *β*-catenin-TCF/LEF1 complex (*: *p* < .05, **: *p* ¡ 0.01, ***: *p* < 0.001, ****: *p* < 0.0001). Scale bars represent 500 µm.

**Figure 5:**
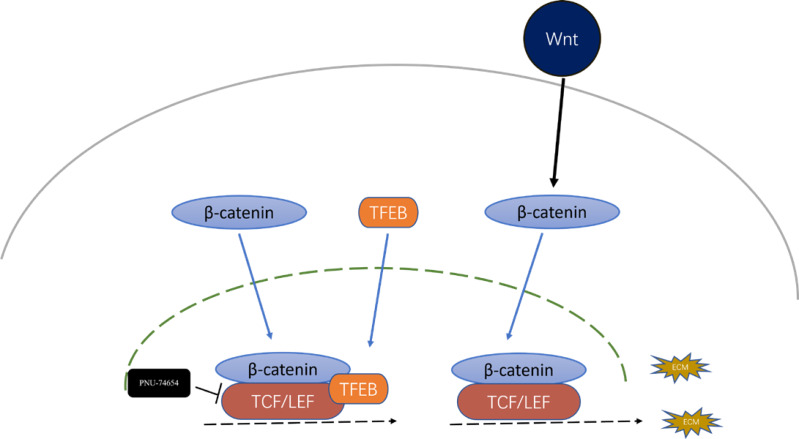
Inhibition effect of TFEB in fibrosis could be mediated by the formation TFEB- *β*-catenin-TCF/LEF1 complex.

Previous studies have provided that the Wnt signaling is involved in cell proliferation and differentiation progress, and is necessary for cardiac myocyte formation (Chen et al., 2001; Moon et al., 2002). Immunofluorescent staining suggested that TFEB didn’t affect the concentration of *β*-catenin. High resolution confocal microscopy analysis showed that colocalization between TFEB and *β*-catenin in the nucleus. By inhibiting the *β*-catenin-TCF/LEF1 complex formation, PNU-74654 decreased the concentration of *β*-catenin in the nuclear (Fig. S7A and Figs. 4A–4B). Lamin-B1 and GAPDH were used as an internal control of nucleus and cytoplasm in Western blot. IF and Western blot showed that TFEB relocated to nuclear in fibrosis model (Figs. S6A–S6B). According to results from CCK8, Transwell, wound healing and western blot assays, the enhancements of cell migration, proliferation and collagen I expression by inhibiting TFEB was prevented by PNU-74654 (Figs. 4C–4F and Fig. S7B). TFEB exerts its anti-fibrotic effect probably by inhibiting Wnt signaling pathway, which has been shown to promote fibrosis (Fig. 5).

## Discussion

Cardiac repair after MI consists of three phases: a pro-inflammatory phase, an anti-inflammatory repair or proliferative phase, and a maturation phase (Ong et al., 2018; Ruparelia et al., 2017a; Westman et al., 2016). This inflammatory process is usually destructive and leads to excessive death of surviving cardiomyocytes, affecting the final infarct size (Zhao et al., 2001). Increased TFEB transcription activated by metformin and cilostazol could protect against I/R injury by regulating autophagy, lysosome, and apoptosis (Li, Xiang & Xu, 2019; Wang et al., 2019). Javaheri et al. (2019) noticed that macrophage-specific over-expression of transcription factor EB (M *ϕ*-TFEB) expression could improve ventricular function after IR injury, and TFEB in macrophages played a role in ventricular remodeling after MI by mediating the inflammatory response. In this study, we verified that TFEB affected left remodeling after MI, demonstrating that TFEB alleviated infarction extension and protected the systolic function of the heart (Figs. 1C–1F). WGA staining showed that TFEB down-regulation was associated with severe cardiac hypertrophy (Fig. 1G). This implies the possibility of using TFEB to protect cardiac function in human MI patients.

As the final stage of MI repair, the maturation phase is associated with remodeling of the ECM which commonly lasts for several months. Scar maturation is a process intertwining the reduction in infarct fibroblast numbers (Frangogiannis, Michael & Entman, 2000), the differentiation of fibroblast into myofibroblast, the apoptosis of activated fibroblasts, the and expression of matrix-specific proteins (Fu et al., 2018). The purpose of scarring is to prevent myocardial rupture and deterioration of partially restricted cardiac function, with thinning and dilation of the infarcted area and hypertrophy of the other areas (Ong et al., 2018). Thus, LV remodeling predicts a poor clinical prognosis. The main pathological features of ventricular remodeling include extensive fibrosis, pathological cardiomyocyte hypertrophy, and cardiomyocyte apoptosis. The balance between excessive synthesis and degradation of myocardial fibrotic collagen is critical for maintaining myocardial ECM homeostasis. Two to four days after injury, fibroblasts activated by the stimulation of inflammatory cytokines began to proliferate and produce ECM (Kurose, 2021). The transformation from fibroblasts to myofibroblasts mainly occurred four to seven days after MI (Fu et al., 2018). Myofibroblasts are characterized by the extensive endoplasmic reticulum, the expression of *α*-smooth muscle actin (*α*-SMA), and the synthesis of matricellular proteins (Hinz, 2010). Thus, we detected the myofibroblasts by *α*-SMA staining. In our study, TFEB inhibited fibroblast differentiation into myofibroblasts as early as three days after MI (Fig. 3A). TFEB inhibited collagen I expression in the fibrosis model *in vivo* (Fig. 3F). Four weeks after MI, TFEB over-expression decreased collagen III synthesis in mice (Fig. 2A). In the context of myocardial fibrosis, TFEB inhibited cell migration and collagen I concentration at the cellular level (Figs. 3D–3F). Unveiling the impacting mechanisms of TFEB in the fibrosis process requires further investigations.

Previous studies have provided that the Wnt signaling is involved in cell proliferation and differentiation progress, and is necesssary for cardiac myocyte formation (Chen et al., 2001; Griffin et al., 2022; Moon et al., 2002; Zhang et al., 2022). In MI model, inhibition of Wnt signaling was shown to reduce collagen concentration and improve cardiac function (Barandon et al., 2011; Fan et al., 2018; Laeremans et al., 2011). The Wnt proteins have been mainly implicated in the promotion of cardiac fibroblast proliferation and collagen expression process (Laeremans et al., 2011).

In recent years, many studies have reported the role of Wnt signaling in cardiac fibrosis in various animal models (Deb, 2014). Classical Wnt/ *β*-catenin signaling leads to epicardial fibrosis in allogeneic heart grafts, and increased activation of *β*-catenin and TCF/LEF is observed in transplanted human hearts (Ye et al., 2013). Acute ischemic cardiac injury can up-regulate Wnt1 expression, which is initially expressed in the epicardium and subsequently expressed by cardiac fibroblasts in the injured area, and Wnt1 induces proliferation of cardiac fibroblasts and expression of profibrotic genes (Duan et al., 2012; Von Gise & Pu, 2012). The use of Dishevelled (DVL) to inhibit the overexpression of GSK-3 *β* and activate canonical and non-canonical Wnt signaling pathways can induce spontaneous myocardial fibrosis and cardiac hypertrophy (Malekar et al., 2010). Meanwhile, Wnt pathway antagonists have also been used to alter the prognosis of fibrosis. secreted frizzled-related protein (sFRP) is a soluble protein with a structure highly homologous to the Fz receptor of Wnt signaling and is a commonly used antagonist of Wnt pathway (Cruciat & Niehrs, 2013; Sklepkiewicz et al., 2015). In myocardial infarct-related studies, inhibition of the Wnt pathway using sFRP1, sFRP2, or sFRP4 was shown to reduce fibrosis and improve cardiac function (Barandon et al., 2011; Fan et al., 2018; He et al., 2010; Laeremans et al., 2011; Matsushima et al., 2010). Mice lacking sFRP1 exhibit increased expression of Wnt ligands, *β*-catenin, *α*-SMA, and collagen (Sklepkiewicz et al., 2015). Expression of sFRP2 in cardiac fibroblasts activates Wnt/ *β*-catenin signaling and promotes proliferation and expression of ECM genes in fibroblasts (Lin et al., 2016). In contrast, SFRP2-deficient mice produced less collagen and had less fibrosis in cardiac fibroblasts after MI (Kobayashi et al., 2009). By inhibiting GSK-3 *β* in cardiac fibroblasts and activating the classical Wnt pathway, fibrogenesis in the infarcted heart can be promoted (Lal et al., 2014). In addition, the development of post-inflammatory fibrosis was successfully prevented by the administration of sFRP2 in a mouse model of autoimmune myocarditis (Blyszczuk et al., 2017). In a model of myocardial fibrosis induced by aortic coarctation, inhibition of *β*-catenin in cardiac fibroblasts reduced interstitial fibrosis without changing the number of activated cardiac fibroblasts (Xiang, Fang & Yutzey, 2017).

Another significant finding of this study is the identification of TFEB as an important regulator of the Wnt signaling. TFEB is a well-known master regulator of autophagy and lysosomal biogenesis processes (Du et al., 2022; Evans et al., 2022; Godar et al., 2015; Ma et al., 2015). In the process of fibrosis modeling, TFEB and *β*-catenin were observed to colocalize within nucleus in fibrosis modeling (Fig. 4A). By inhibiting the *β*-catenin-TCF/LEF1 complex formation, PNU-74654 decreased the concentration of *β*-catenin in the nuclear (Fig. 4B). Our findings indicated that the concentration of *β*-catenin was not influenced by TFEB. Moreover, PNU-74654 was observed to counteract the increase in cell migration, proliferation, and collagen I expression that resulted from the inhibition of TFEB (Figs. 4A–4F). The nuclear localized TFEB forms a TFEB- *β*-catenin-TCF/LEF1 complex to induce the transcription of genes, that being distinct from previously known regulated by the *β*-catenin-TCF/LEF1 complex (Kim et al., 2021).The very first possibility is that the inhibition effect of TFEB in fibrosis was mediated by the formation TFEB- *β*-catenin-TCF/LEF1 complex, which changed the gene expression profile of *β*-catenin (Fig. 5). It is possible that the influence of TFEB on fibroblasts is mediated *via* the Wnt pathway. Our study provides evidence that TFEB’s anti-fibrotic activity is likely achieved through the suppression of the Wnt signaling pathway, a mechanism known to foster fibrosis However, more studies may be needed to clarify other pathways through which TFEB may influence.

##  Supplemental Information

10.7717/peerj.15841/supp-1 Supplemental Information 1 Checklist

10.7717/peerj.15841/supp-2 Figure S1 Image of MI mouse hearts under natural light. Image of mouse hearts under natural light four weeks after MI

10.7717/peerj.15841/supp-3 Figure S2 Mice echocardiography and the qualified EF ratios in each group

10.7717/peerj.15841/supp-4 Figure S3 TFEB impacts myocardial hypertrophy induced by MI modeling (A) The myocyte areas stained by the wheat germ agglutinin (WGA). Scale bars represent 50 µm. (B) Myocyte areas at different time points after MI modeling. (C) Myocyte areas at different times after MI modeling in different groups (qualified from the WGAs staining) (*: *p* < 0.05, **: *p* < 0.01). Scale bars represent 50 µm.

10.7717/peerj.15841/supp-5 Figure S4 Viral transfection altered TFEB expression and nuclear translocation (A) Confocal image of the Immunofluorescence staining of TFEB (yellow) of mouse heart three days after MI modeling. Scale bars represent 50 um. (B) The expression of TFEB of mouse heart three days after MI modeling was detected using Western blotting.

10.7717/peerj.15841/supp-6 Figure S5 Wound healing showed cell migration rate (4X)

10.7717/peerj.15841/supp-7 Figure S6 TFEB relocated to nuclear in fibrosis model (A) Immunofluorescence staining of TFEB (GFP) of the CFs co-incubated with TGF- *β*1 (5 ng/mL) for 12 hours. Scale bars represent 1mm. (B) Western blot of TFEB expression in the nucleus and cytoplasm in CFs.

10.7717/peerj.15841/supp-8 Figure S7 TFEB made connection with Wnt pathway by *β*-catenin-TCF/LEF1 complex (A) Immunofluorescence staining of *β*-catenin (red) of the CFs co-incubated with PNU-74654 (100 ng/mL) was used to for 12 hours. Scale bars represent 100 um. (B) Wound healing showed CFs migration rate with PNU-74654 (100 ng/mL) was used to for 12 hours (4X).

10.7717/peerj.15841/supp-9 Supplemental Information 9 Gel

10.7717/peerj.15841/supp-10 Data S1 Raw data

## Abbreviations

AAV9: Adeno-associated virus 9
Ad: adenovirus
*α*-SMA: *α*-smooth muscle actin
CCK8: cell counting kit-8
ECM: extracellular matrix
EDU: Cell Proliferation EdU Image
HF: heart failure
I/R: ischemia/reperfusion
LAD: anterior descending branch of the left coronary artery
LV: left ventricular
MI: myocardial infarction
TFEB: transcription factor EB
WGA: Wheat germ agglutinin

## Additional Information and Declarations

### Data Availability

The following information was supplied regarding data availability: Data is available at OSF: Liu, Cong. 2023. “Raw Data.” OSF. May 24. doi: 10.17605/OSF.IO/X7QPV.
