# Supplementary figures and images for: Transcription factor EB (TFEB) improves ventricular remodeling after myocardial infarction by inhibiting Wnt/β-catenin signaling pathway
Source: PeerJ. 2023 Aug 18;11:e15841. doi: 10.7717/peerj.15841 (PMC10441526; doi:10.7717/peerj.15841)

AAV9-NC

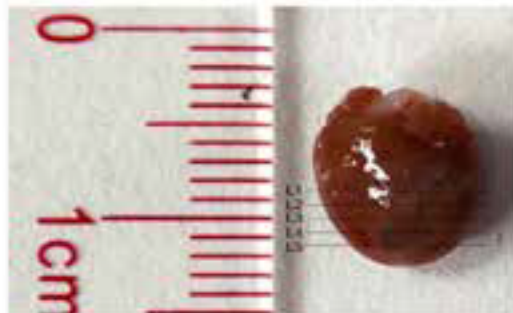

AAV9-TFEB

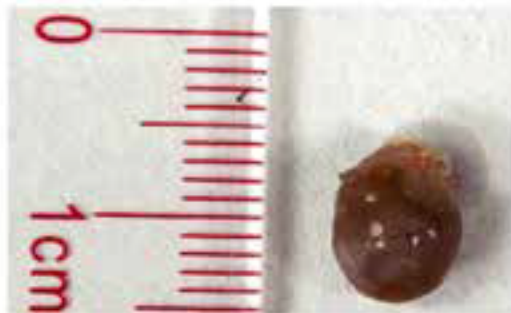

AAV9-shNC

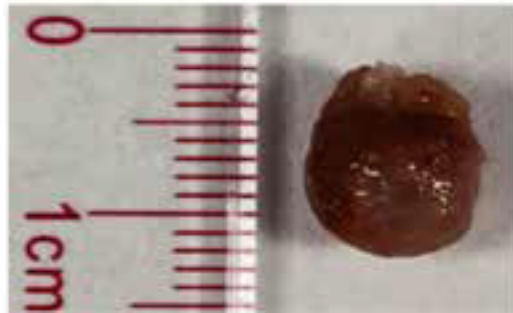

AAV9-shTFEB

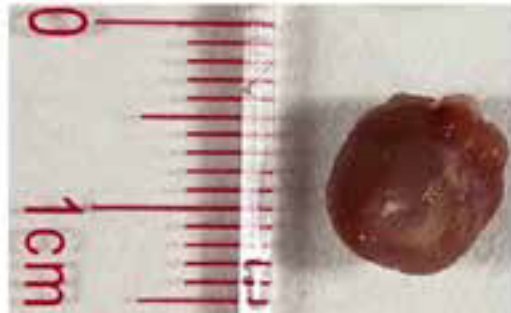

Supplement: Figure S1 [file peerj-11-15841-s002.pdf]

**A**

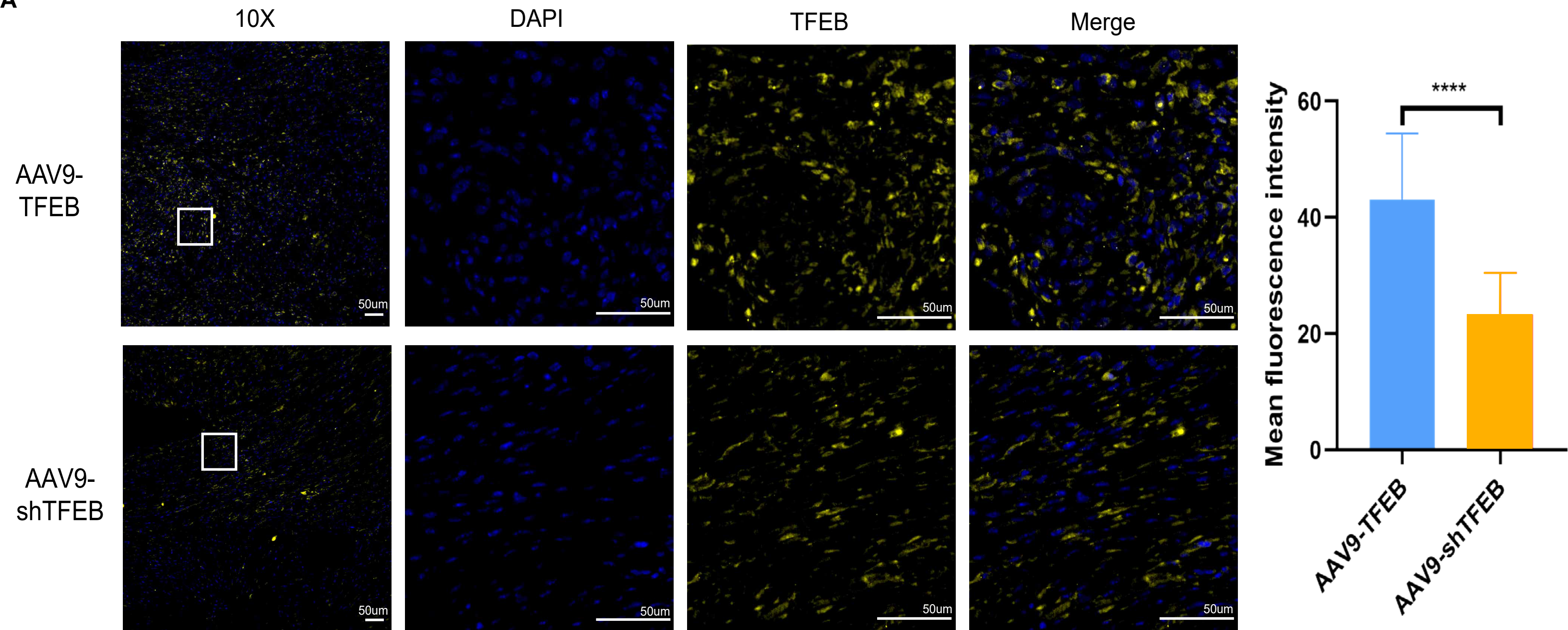

**B**

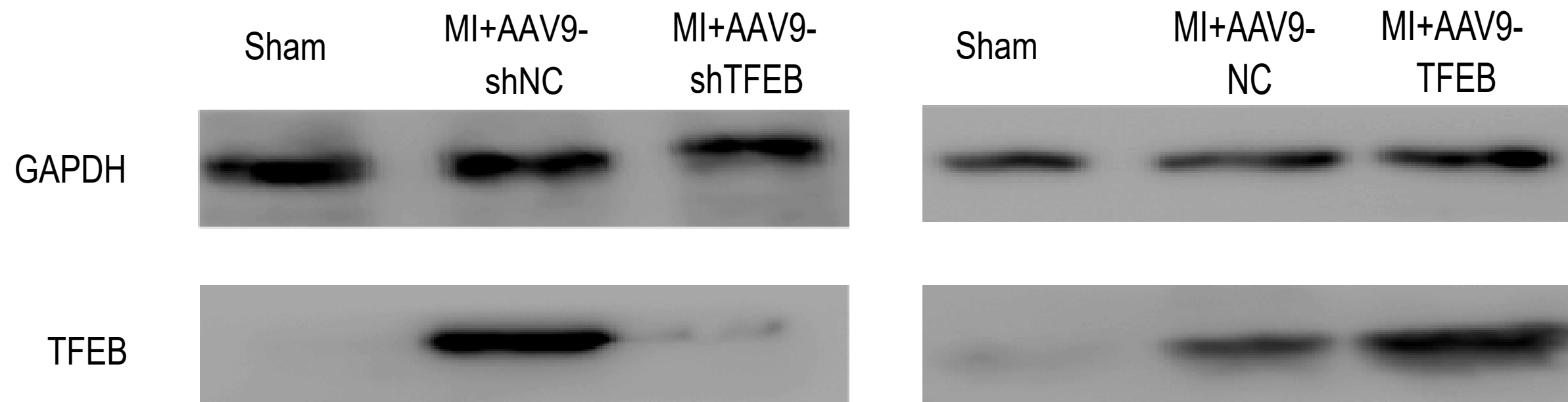

Supplement: Figure S2 [file peerj-11-15841-s003.pdf]

3W

4W

AAV9-NC

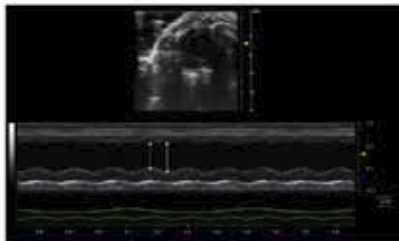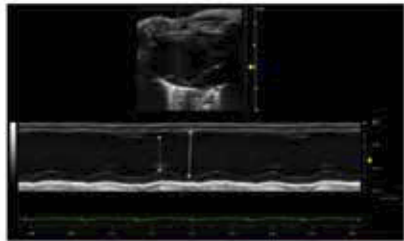

AAV9-TFEB

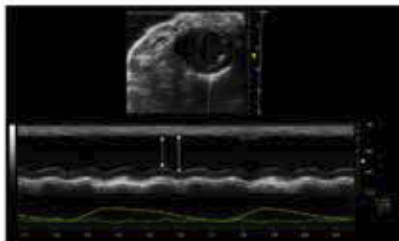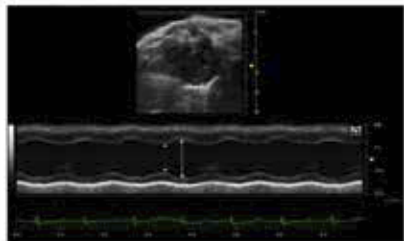

AAV9-shNC

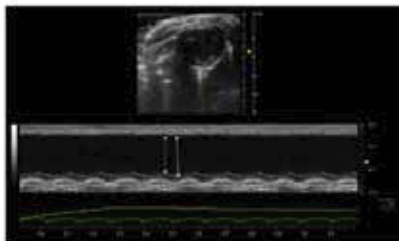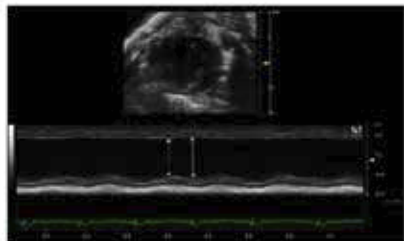

AAV9-shTFEB

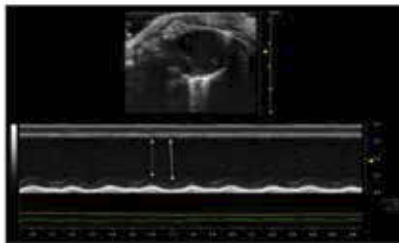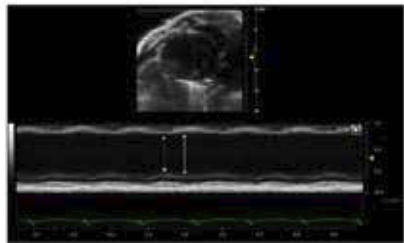

Supplement: Figure S3 — (A) The myocyte areas stained by the wheat germ agglutinin (WGA). Scale bars represent 50 µm. (B) Myocyte areas at different time points after MI modeling. (C) Myocyte areas at different times after MI modeling in different groups (qualified from the WGAs staining) (*: p < 0.05, **: p < 0.01). Scale bars represent 50 µm. [file peerj-11-15841-s004.pdf]

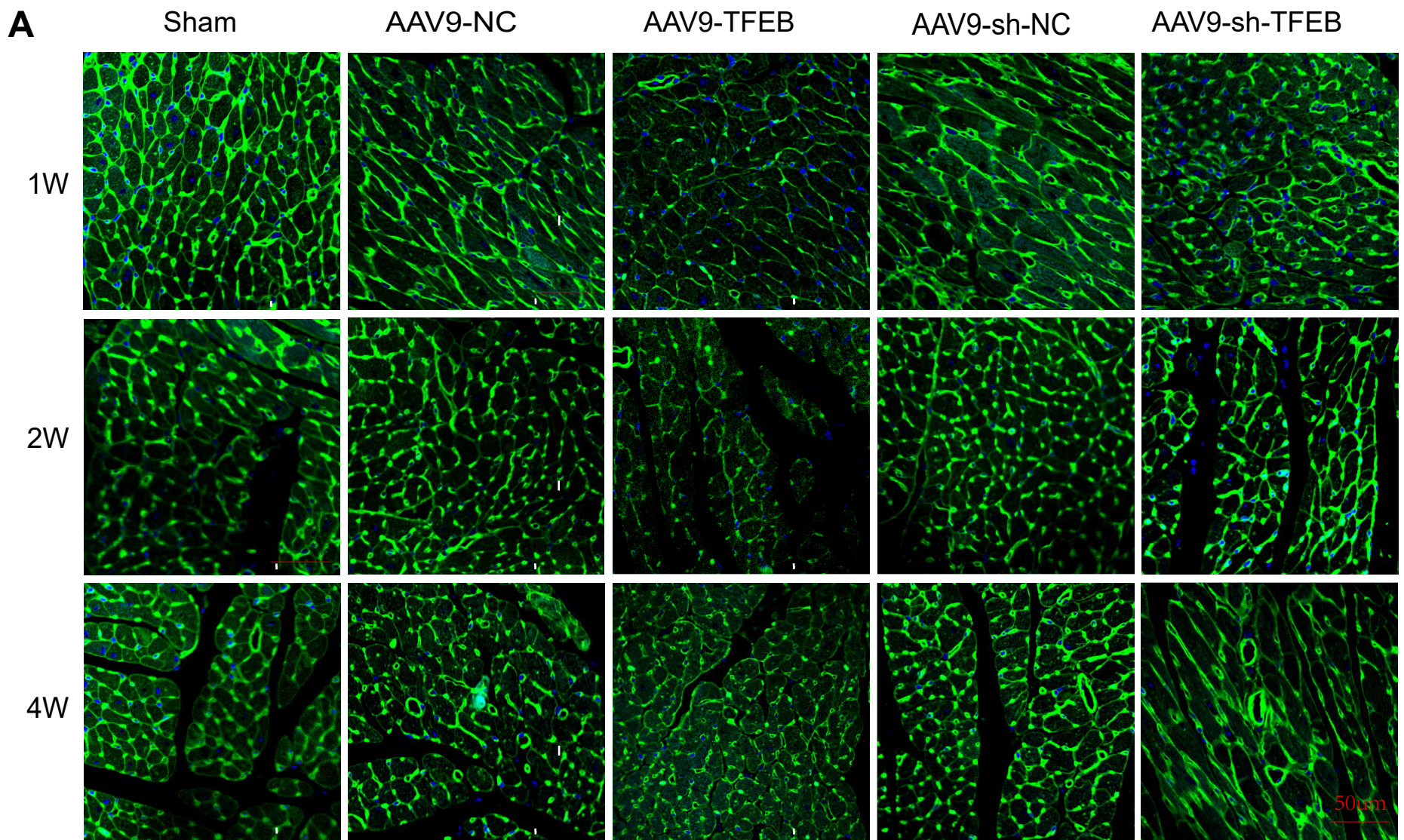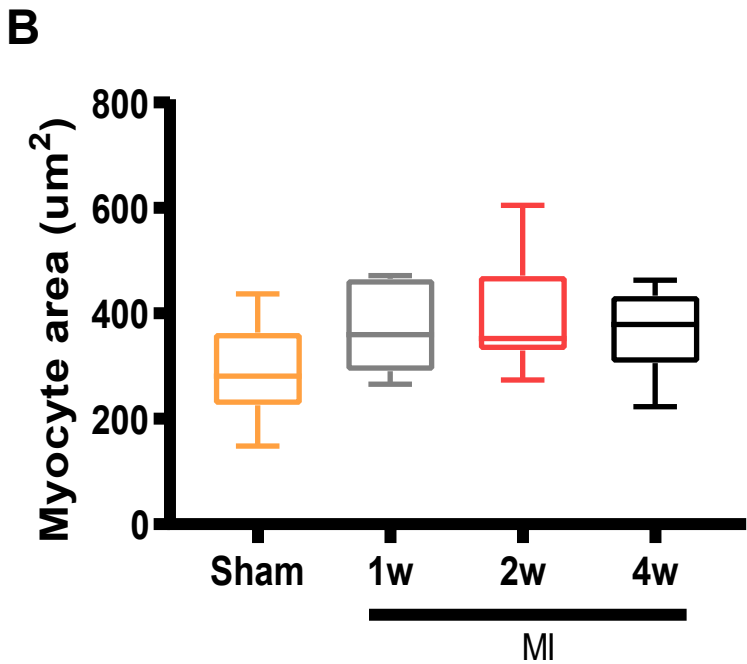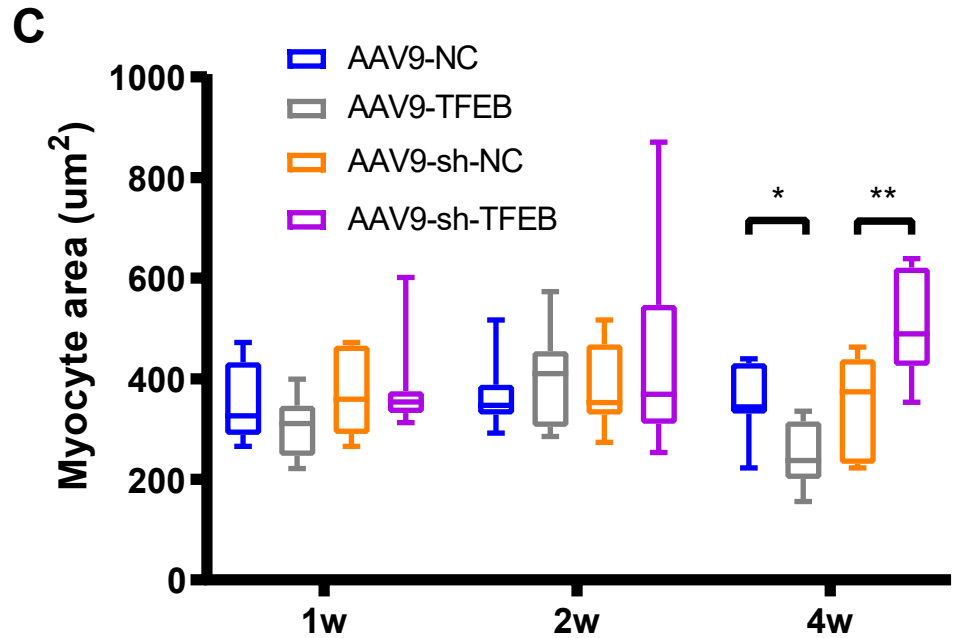

Supplement: Figure S4 — (A) Confocal image of the Immunofluorescence staining of TFEB (yellow) of mouse heart three days after MI modeling. Scale bars represent 50 um. (B) The expression of TFEB of mouse heart three days after MI modeling was detected using Western blotting. [file peerj-11-15841-s005.pdf]

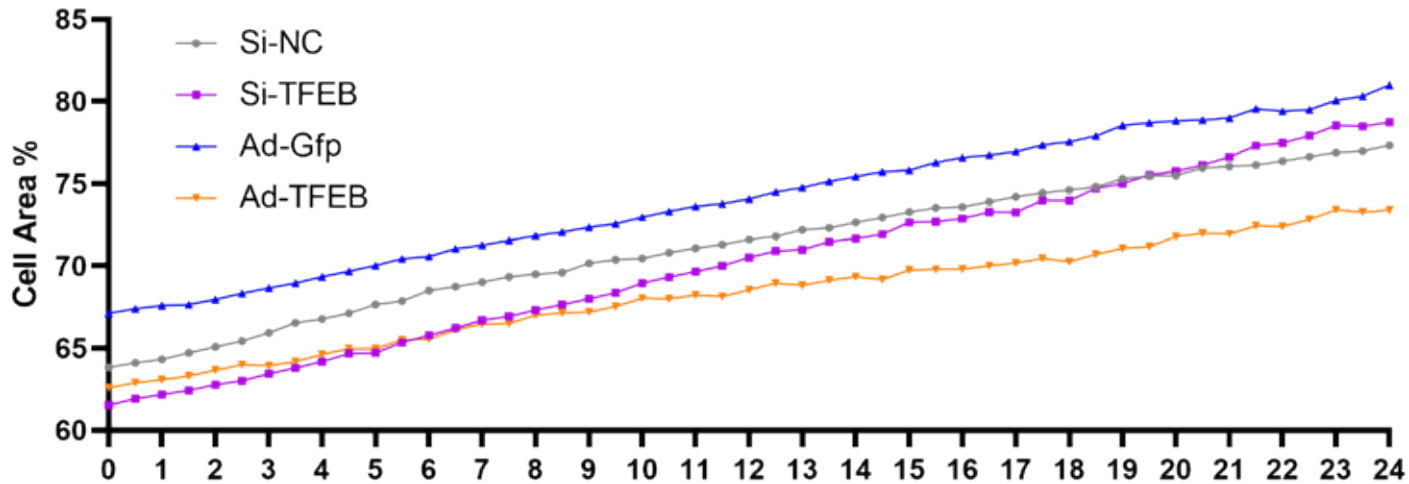

Supplement: Figure S5 [file peerj-11-15841-s006.pdf]

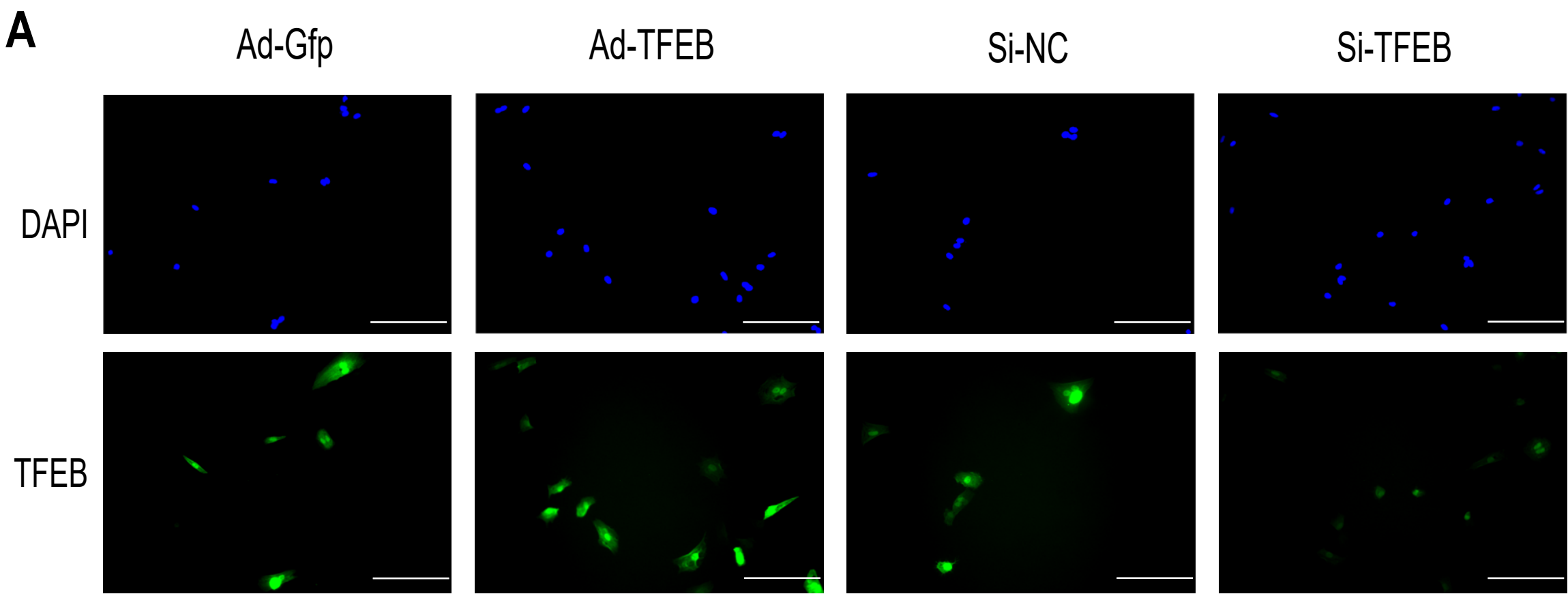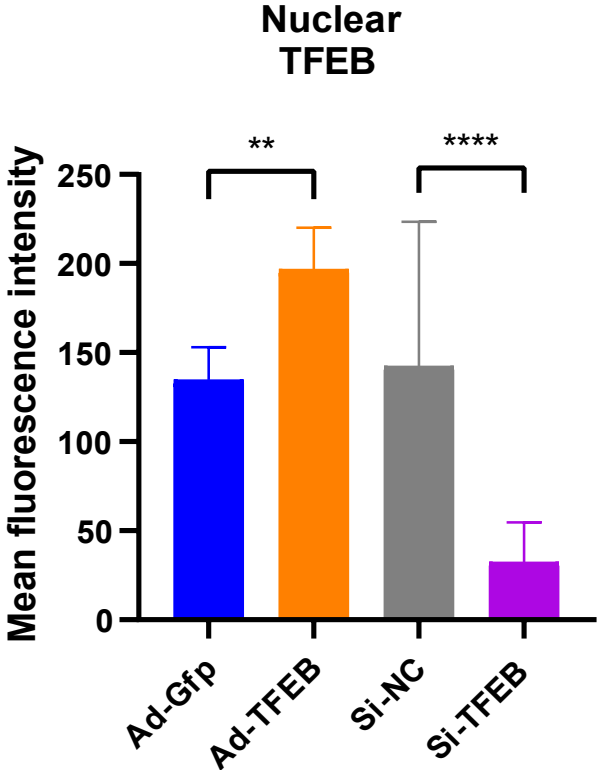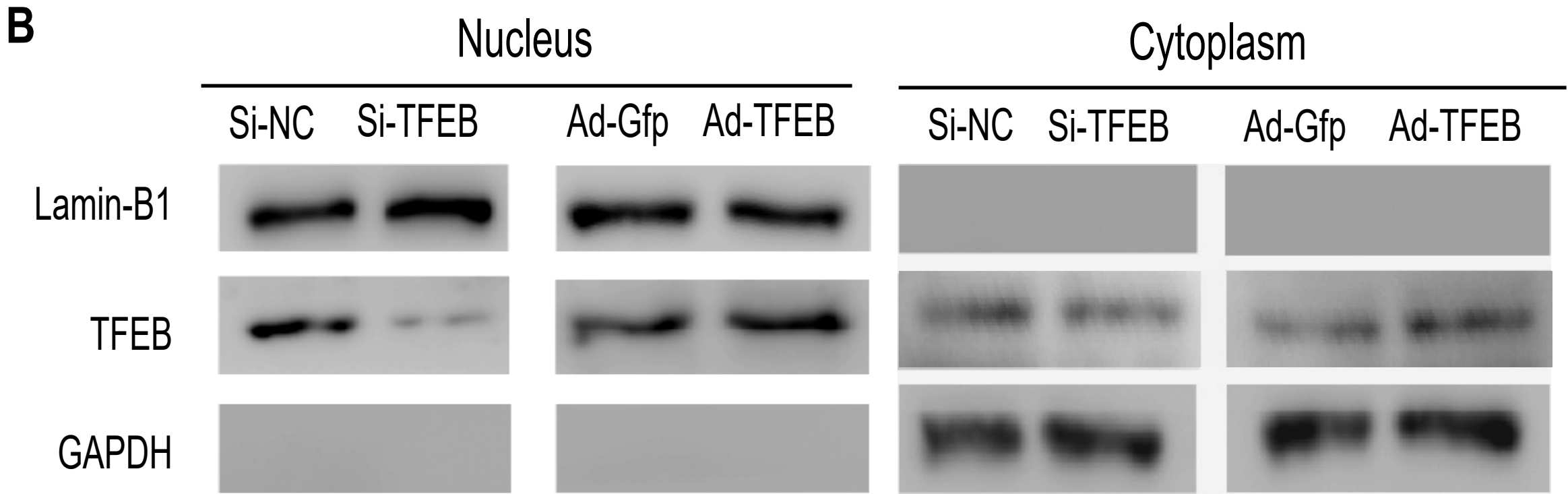

Supplement: Figure S6 — (A) Immunofluorescence staining of TFEB (GFP) of the CFs co-incubated with TGF- β1 (5 ng/mL) for 12 hours. Scale bars represent 1mm. (B) Western blot of TFEB expression in the nucleus and cytoplasm in CFs. [file peerj-11-15841-s007.pdf]

**A**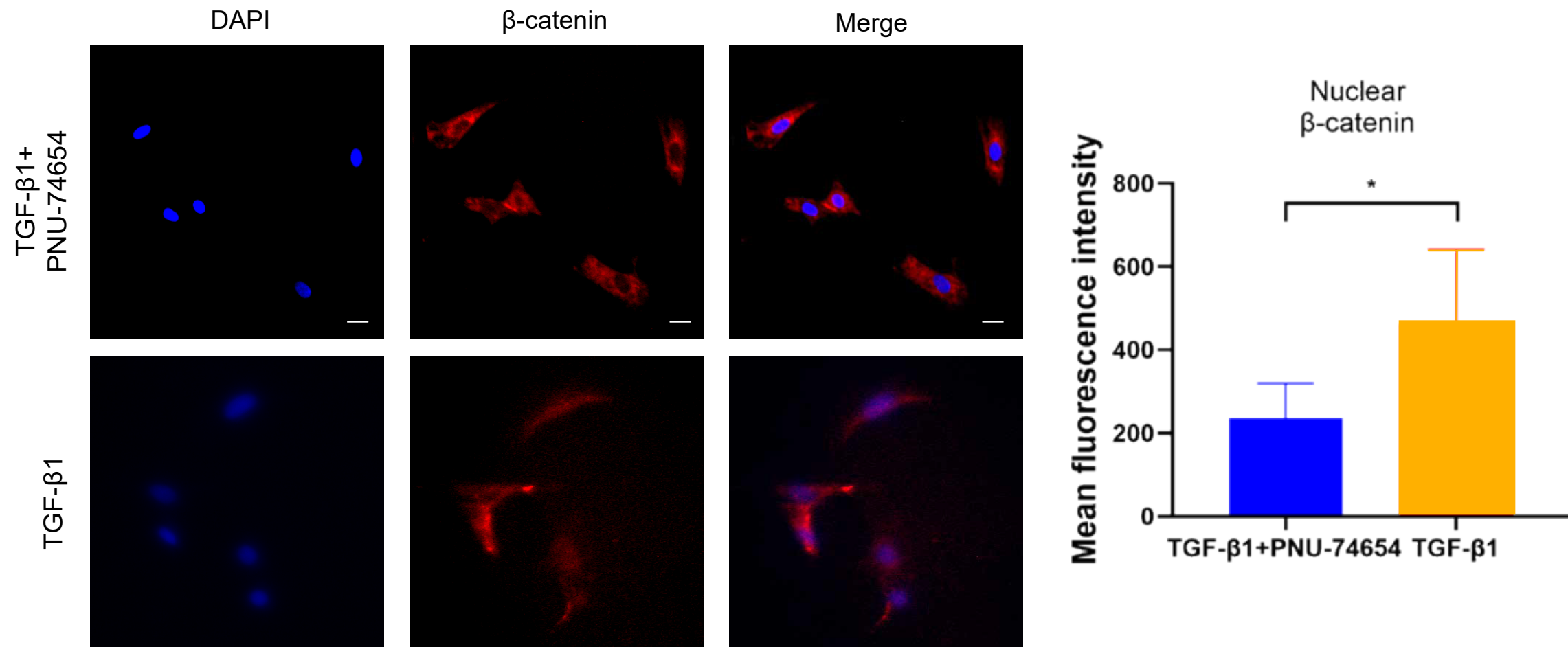**B**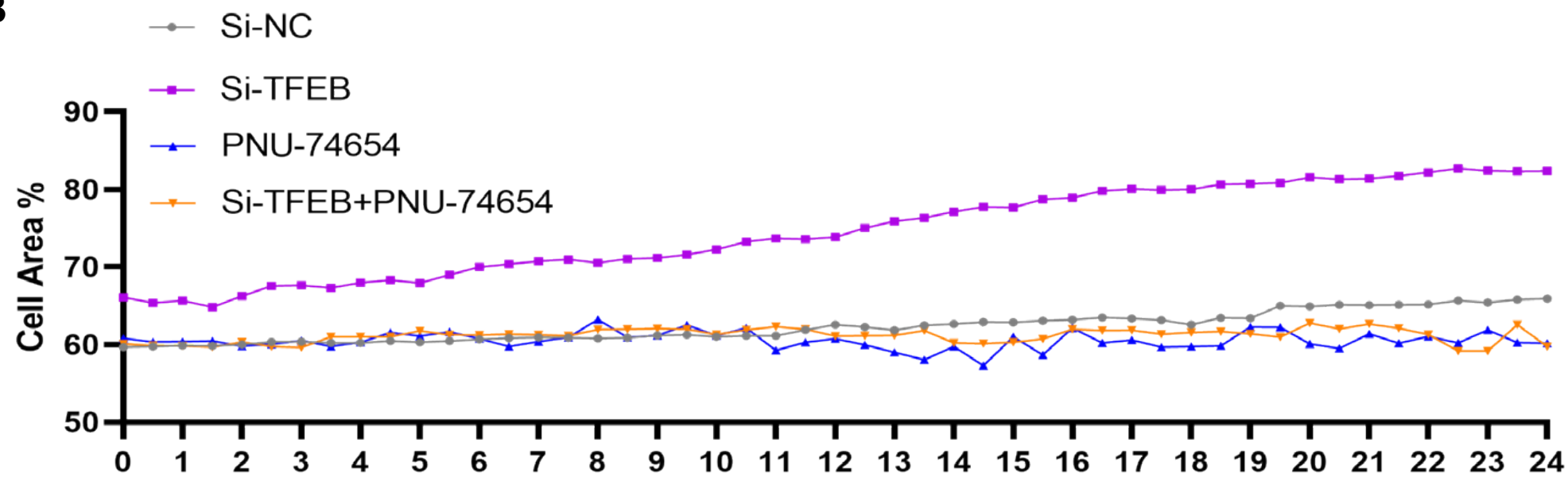

Supplement: Figure S7 — (A) Immunofluorescence staining of β-catenin (red) of the CFs co-incubated with PNU-74654 (100 ng/mL) was used to for 12 hours. Scale bars represent 100 um. (B) Wound healing showed CFs migration rate with PNU-74654 (100 ng/mL) was used to for 12 hours (4X). [file peerj-11-15841-s008.pdf]
