# Supplementary material for: Transcription factor EB (TFEB) improves ventricular remodeling after myocardial infarction by inhibiting Wnt/β-catenin signaling pathway
Source: PeerJ. 2023 Aug 18;11:e15841. doi: 10.7717/peerj.15841 (PMC10441526; doi:10.7717/peerj.15841)
Supplement: Supplemental Information 9 [file peerj-11-15841-s009.zip › gel - ╕▒▒╛.pdf]

Figure3.

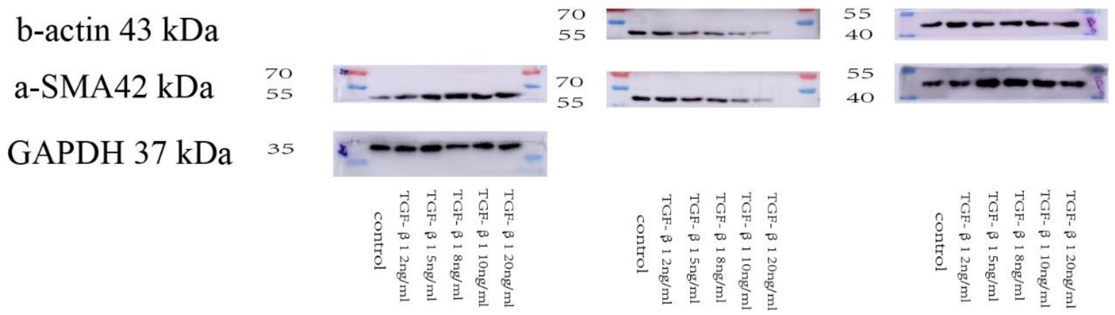

Effects of TGF-β1 of different concentrations and incubation time on the expression of α-SMA was detected using Western blotting.

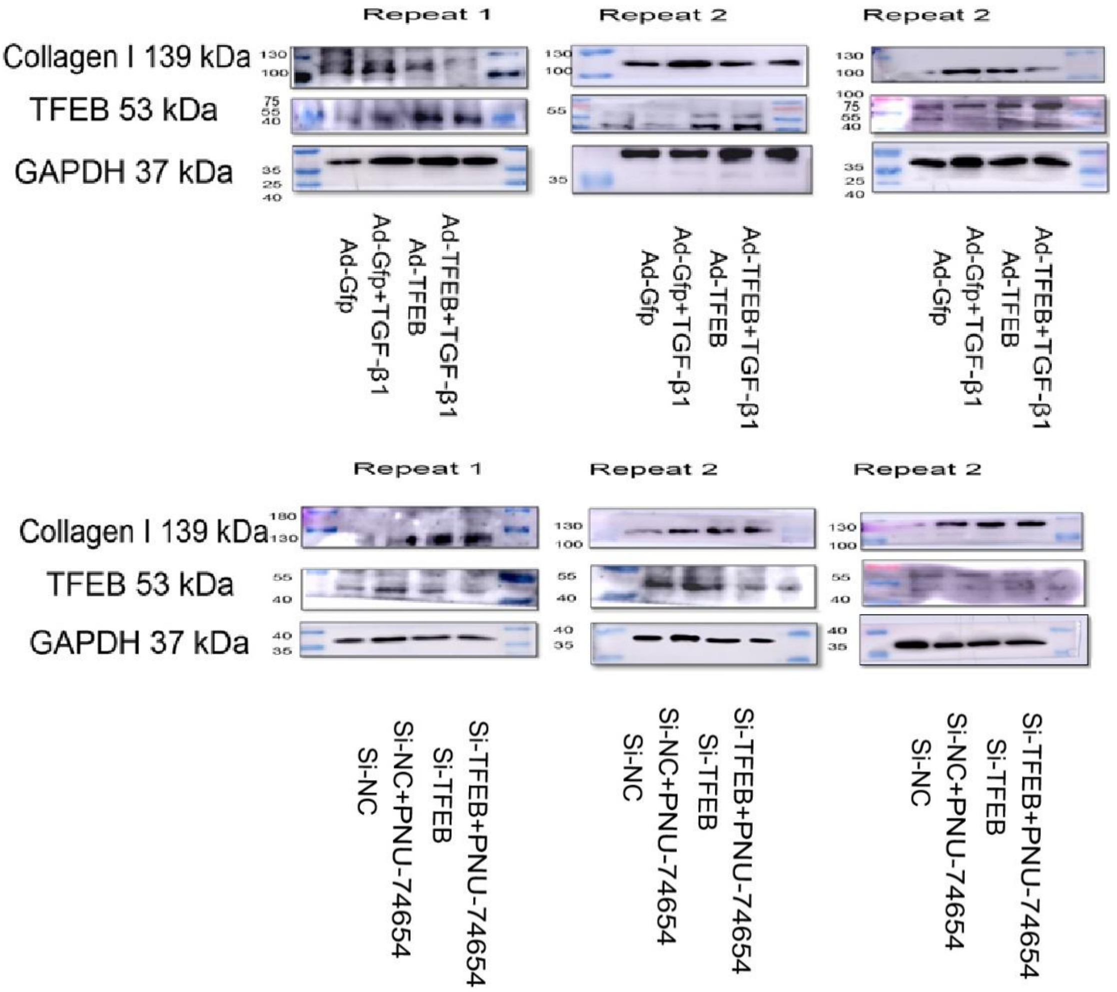

The expression difference of Collagen I of CFs co-incubated with TGF-β1 (5 ng/mL) for 24 hours was detected using Western blot.

Figure4.

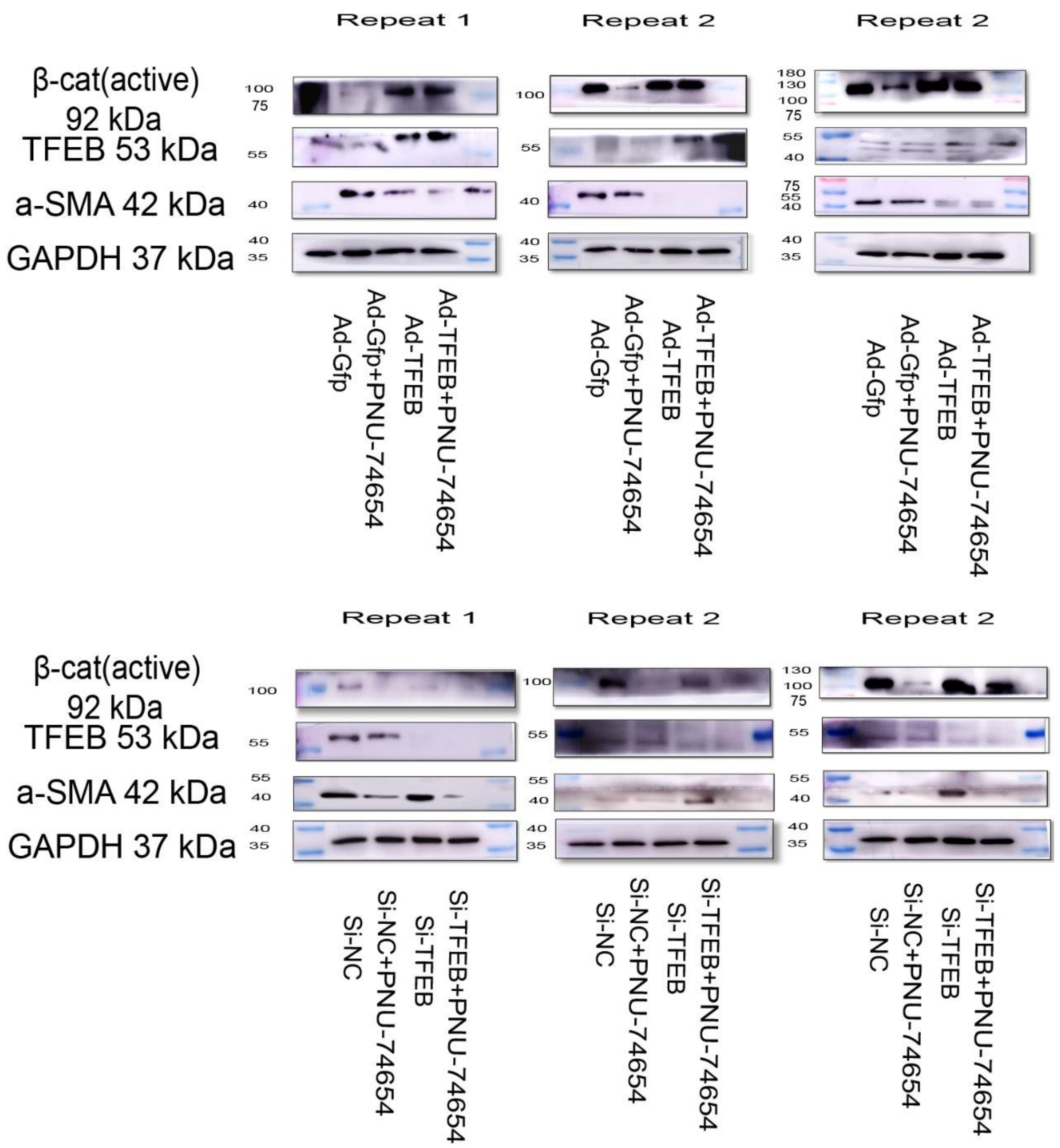

The expression of α-SMA was detected using Western blotting. PNU-74654 (100 ng/mL) was used to for 12 hours to inhibit β-catenin-TCF/LEF1 complex.
